# Cascading collapse of online social networks

**DOI:** 10.1038/s41598-017-17135-1

**Published:** 2017-12-01

**Authors:** János Török, János Kertész

**Affiliations:** 1 Center for Network Science, Central European University, Nádor u. 9, H-1051 Budapest, Hungary; 2 Department of Theoretical Physics, Budapest University of Technology and Economics, H-1111 Budapest, Hungary

## Abstract

Online social networks have increasing influence on our society, they may play decisive roles in politics and can be crucial for the fate of companies. Such services compete with each other and some may even break down rapidly. Using social network datasets we show the main factors leading to such a dramatic collapse. At early stage mostly the loosely bound users disappear, later collective effects play the main role leading to cascading failures. We present a theory based on a generalised threshold model to explain the findings and show how the collapse time can be estimated in advance using the dynamics of the churning users. Our results shed light to possible mechanisms of instabilities in other competing social processes.

## Introduction

Growth as a main route to the formation of complex networks have been studied in great detail^1–4^. Much less effort has been devoted to the understanding of the complex process of the decline of networks^5^. In this context the effect of random failures and intentional attacks^6–9^ has been investigated and recently the enhanced vulnerability and cascading breakdown of interdependent networks^10^ or k-core percolation^11^ were shown. The disintegration of real networks is usually a consequence of an interplay between endogenous and exogenous factors and its understanding is of major interest for a series of important questions like the decay of living organisms, the disintegration of social networks or the loss of market share in economic competition.

Social contagion^12,13^ like adoption of opinions, behavioural patterns, emotions^14–17^ or innovations can be considered as growth of a network of adopters on the top of an underlying network namely that of social interactions. Under some circumstances this process is surprisingly rapid. Social pressure plays a pivotal role in this context: People are influenced in their decisions by the opinions of their peers^14,15^. This effect is captured in the so called threshold models^18–20^, which assume that a person becomes adopter, when the ratio of her already adopting, related peers have reached a critical level characteristic to her sensitivity.

The above spreading processes are constructive in the sense that as a result a network of adopters emerges. The study of such processes have been boosted by the abundance of information communication data^21–27^ and has lead to a deeper understanding of how adopter networks emerge due to complex social contagion^17,19,21,25,28–32^. However, there can be an opposite process, when people forming a network of users of some technology or service leave this network. The reason can be getting uninterested in the service or churning to another provider^33^. Are there collective effects in this process as well, with the possibility of a dramatic collapse of the entire adopter network? The first aim of our paper is to use the data to show that this can indeed be the case and to present a model, which is able to describe the time dependence of the decomposition of such a networks. The second aim of this paper is to verify the model if it is able to predict in advance the collapse of a site.

After the success of Facebook (https://facebook.com) a particularly keen competition has developed among Online Social Networks (OSN-s) resulting in the defeat of a number of earlier popular sites. We will focus in this study data on *iWiW* ^34^, which used to be the most successful Hungarian OSN. Results will also be shown for a much smaller OSN the Gowalla available from Large Stanford Dataset Collection^35^.

## Empirical Results

Here we present results for iWiW^34^, the Hungarian OSN, which was active between 2004 and 2013 and was in the list of the top three most popular sites in the country for 2007–2011^36^. The details of the data are presented in the section Materials and Methods. We carried out the same analysis for another social network site: Gowalla, (see the section Other Empirical Networks in Supporting Information).

**Figure 1:**
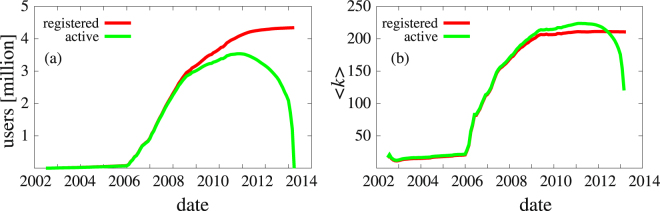
Timeline of properties of iWiW. (**a**) The number of registered users (red), the number of active users (green), (**b**) average degree of the nodes, all registered (red), only among active (green).

**Figure 2:**
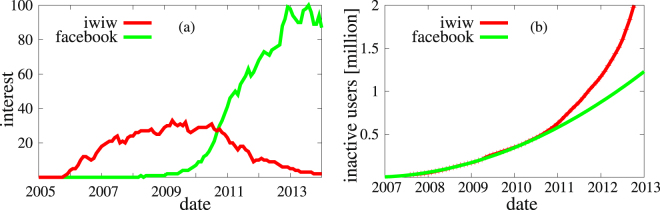
Time evolution of interest for social network sites in Hungary and the inactive users on iWiW. (**a**) Google trends of iWiW and Facebook for Hungary. (**b**) Number of users who became inactive in until a given month. The green line is a parabola fit in the form of 210 *m* ^2^, where *m* is the number of months after July 2006. Before August 2010 this gives a perfect fit indicating a linearly increasing churning rate. After that time the rate grows super linearly.

The nodes of the network are the users of the service and links are the mutually acknowledged connections (“friendships”). Fig. 1 shows the time evolution of the number of users of iWiW as well as the average degrees for all and for the active nodes where a node is considered to be inactive after its last login. After an almost latent period the number of users started growing rapidly early 2006 (for reasons see Materials and Methods section) and until mid 2007 the number of leavers remained negligible. It is interesting to note that even though in Fig. 2(a) Google trends shows that already in September 2010 the number of hits for Facebook was higher than that for iWiW, the number of active users of iWiW still increased for a few more months. The average degree of all users remained constant after 2009 but the average degree of the active users increased slightly till mid 2011 as shown in Fig. 1(b), indicating that less embedded users left first.

Figure 2(b) shows the cumulative number of users who became inactive in a given month. Some users left the service already as early as 2007 and from that point on the cumulative number of inactive users increases quadratically, meaning that the number of users leaving the service each month increases linearly with time. This trend remained characteristic for the system for the period 2007–2010. The observed linear increase in the rate of users leaving the service is the result of an interplay between the slowly growing popularity of Facebook and the increasing user pool.

Around the end of 2010 the number of users leaving iWiW started to accelerate, which turned into a dramatic loss and lead finally to the collapse of the entire OSN. The turning point in the history of iWiW can be seen on the Google trends (Fig. 2(a)) where the popularity of Facebook in Hungary started to increase rapidly while the popularity of iWiW to decline, at the end of 2010. The question is whether the dramatic increase in churning can be simply explained by the increase in the interest of people in the competing service or is there a collective effect. We will show that the latter is the case.

**Figure 3:**
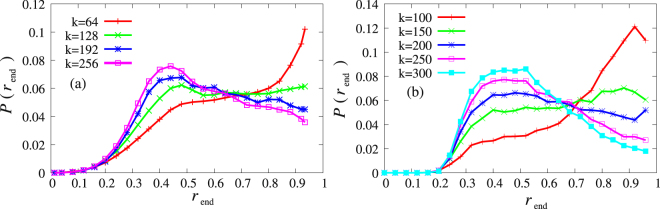
Fraction of active friends at the time of last login. (**a**) Distribution of the fraction of active acquaintances at the time of the last login of users for four different user degrees in iWiW. Note that the absence of values less than *r*_end_ ≃ 0.3 is due to the fact that our database was truncated when 70% of the users left. (**b**) Same plot for the model using an extended network with *N* = 10^4^ agents and 〈*k*〉 = 220.

In order to study the above question we calculated the fraction *r*_end_ of the active acquaintances of a user at the time of her last login with a week overhead. The results about the distribution of *r*_end_ are shown in Fig. 3(a) for users with different degree.

The distribution of the fraction of active friends at the time of last login depends in a specific way on the number *k* of friends of the users. While the distribution is for all *k*-values smooth and spreads over almost the whole range of *r*_end_, the position of the maximum jumps from ~0.9 for *k* < 130 to 0.4−0.5 for *k* ≥ 130 (see Supporting information for more details). This indicates the following mechanism: For all *k* values there are persons, who leave the OSN just due to the exogenous input–those, who have an active friend ratio close to 1 are certainly such users as they have not been influenced by the churning of their friends. Then, there are persons, who leave, when a considerable part of their partners have left. We can expect that the level of embeddedness into the OSN as measured by the number of friends has an impact on which of these factors is dominant. The users with the maximum close to 1 are mainly those with low embeddedness in (or affinity to^37^) the service as they have only relatively low number of friends in the OSN. On the other hand users with high affinity have many friends on the OSN, and they stick to the service as long as they can reach a good fraction of their friends. The data suggests that for the latter group the maximum of the distribution of active friends at the time of churning is around 45%. In the early period (mid 2007–end 2010) churning was mainly due to independent decisions triggered by some exogenous input or loss of interest. The deviation from the smooth, linearly increasing rate indicates the appearance of the collective effects.

The mechanism of the sharp transition from one position of the maximum to the other one can be understood such that the broad distributions result from two main processes: One with a maximum close to 1 and the other at around 0.5 and the weights of these get shifted from the former to the latter one as the degree increases (see Fig. 3).

**Figure 4:**
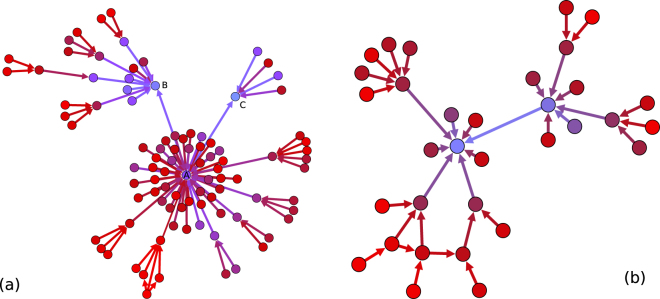
Sample cascades. Arrows mark links between users who have acknowledged each other as friends and the user at the tail of the arrow left not more than a week before the one at the head. Colour changes from red to light blue as time passes. (**a**) cascade based on iWiW data, note that after the leave of many friends of A, it also left the site which then triggered the leave of users B and C. The time evaluation of a cascade is shown in a movie in the supplementary information; (**b**) model example cascade.

The existence of thresholds in the decision to leave the service may lead to collective phenomena of avalanches or cascades. Indeed, we find such cascades in the data; for an example see Fig. 4(a). A user was chosen who left the service in March 2011. Arrows are plotted between friends if they left the service in the given order (tail first) within one week. Users were traced back up to a two months period with the above condition unfolding this way the process backwards. There are users, who seem to leave the OSN without any endogenous trigger in this representation. On the other hand, there are clear examples of cascades already on this one week scale, where users get inactive after many of their friends got inactive. Thus the mechanism resulting finally in a complete collapse of the OSN is as follows: At the beginning people leave spontaneously the service due to lack of interest or finding another provider more satisfactory. These are usually individuals, who are not bound by many ties to the network. As the interest in the competing service rises (cf. Fig. 2(a)) the number of such churners increases such that the threshold level of persons with high degree is reached, releasing further departures from the service and, occasionally, cascades. However, the cascades are finite and need to be further triggered by those who churn “spontaneously”, i.e., due to exogenous influence. This is similar to the situation recently observed for innovation spreading^26,32^, which gives inspiration to modelling our empirical findings.

**Figure 5:**
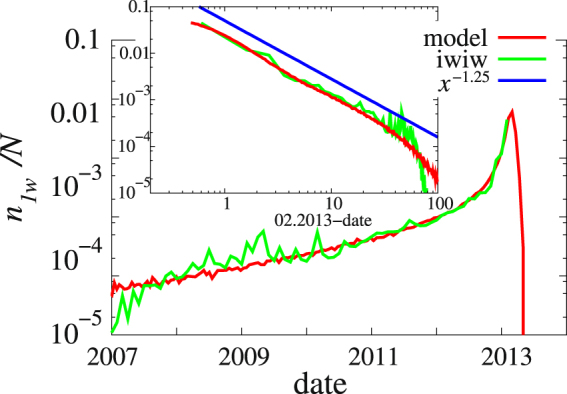
Time evolution of churning users. The average number *n*_1*w*_ of friends churning during the week before the last login of a user plotted against the time difference to the time of the maximal user loss in case of the model and to the date February 2013 in case of iWiW. The slope of the straight line is ~1.25. The inset shows the same data with the *x* axis indicating the number of months after 01.2007.

We measured the average number of friends of a user churning during the last 1, 2, or 4 weeks before she left (denoted by *n*_1*w*_, *n*_2*w*_, *n*_4*w*_ respectively). Since apart from a constant shift no important difference was found between these quantities only *n*_1*w*_ is plotted in Fig. 5, where we see the following scenario: Users start to leave the OSN as early as in 2007 and has a strongly fluctuation period around *n*_1*w*_ ≈ 2 10^−4^, when the OSN is at its best. Beginning late 2010 *n*_1*w*_ starts to increase rapidly and finally it shoots up at early 2013, which can be described as a power law with an exponent as (*t*_*c*_ − *t*)^−1.25^ with *t*_*c*_ = February 2013 (see inset of Fig. 5). We assume that this power law divergence is a sign of the cascading collapse and can be used to determine the date of the collapse beforehand.

A natural question to ask is whether there are other early signatures of the global breakdown. For example, the statistics of newcomers and of activity periods lead to interesting observations about the healthiness of an the online service Myspace and other OSN-s^14,15^. Here we analyse these two quantities as possible indicators for the collapse of iWiW.

**Figure 6:**
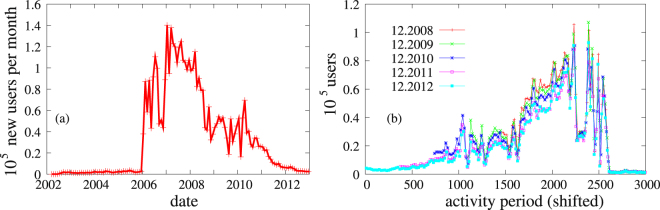
Time evolution of the new users and the activity period of the active users. (**a**) The number of new users per month versus time. (**b**) The histogram of the activity period of the active users. Each bin represents 20 days. The curves were shifted successively by one year so that they would fall upon each other if there was no change. The last curve 12.2012 was multiplied by a factor of 2 for easier comparison with the previous ones.

In Fig. 6(a) the number of new users per month is plotted. One can easily recognise the commercialisation at the beginning of 2006, the big capacity problem in September 2016. There is a big drop in the number of new users in middle 2008. This is related to the big crisis in Hungary. According to the data of Worldbank^38^ the ratio of internet subscriptions increased only 1% in this year compared to the 8% of the previous one. From this period on the number of new users is less than half then previously. This period with 40–60 thousand new users per month lasts till the end of 2011, when it slowly decreases to 0. Obviously the big collapse of the site starts at around this point, but this curve does not allow for an early recognition of this event.

We have seen in Fig. 3 that those with low degree leave the site first. In some online social sites the newcomers replace the old ones (see e.g. gowalla) which keeps the site alive. This hypothesis was tested on iWiW by measuring the distribution of the activity period of the active users which is shown in Fig. 6(b). The curves prior to 2012 were shifted by the number of days between the sample periods, so they fall on top of each other if old users do not leave. Indeed we can see that especially the part of the histograms for large activity period looks very much time independent. Even the big dip due to capacity problems at the end of 2006 is the same on all curves. Note that the last curve of 12.2012 was multiplied by a factor of 2 for better comparison, because of the low number of active users by that time. Still this curve looks very much like the others.

The only difference we can see is that the last part of the older curves are always higher which means that those who did not like the site left not long after registering (most probably with low degree) but the ones who stayed for a while remained almost to the end of the site. The match by a factor of two between 12.2012 and 12.2011 indicates that the activity period was not a prime reason for staying or leaving at the end, the collective motion indicated by the peak in *r*_end_ seems more important.

## Generalised threshold model and simulations

In the following we construct a model for the collapse of OSN-s, which incorporates both exogenous and collective effects on churning. The parameters of the model will be adjusted to the iWiW data.

In order to keep the underlying structure simple, the model is defined on a random network where 40% of the links are created in an uncorrelated random way and the rest is generated by placing random triangles, as high clustering is typical for social networks^39,40^. Finally we have a random network with *N* nodes and an average degree of 〈*k*〉. At the beginning all nodes are part of the OSN. In the Supplementary Information we present results using part of iWiW network as underlying structure.

Social contacts have different intimacy levels building a hierarchy of concentric layers around the persons in their egocentric networks^41^. It is natural to assume that only the set of closest friends matter in the decision of well embedded individuals about staying with the service or leaving it. Therefore we will consider only a network with reduced average degree, which is considered as a parameter.

For the endogenous, collective effects we implement a threshold mechanism^18,19^: Whenever the fraction of active friends of a node drops below its threshold *λ* the node will feel inclined to leave the OSN. This does not happen immediately but with a rate 1/*τ*, so *τ* is the timescale of leaving the OSN after the threshold condition is fulfilled. All nodes are given a predefined uncorrelated threshold value *λ* = 0.5 ± 0.2 with uniform variations.

The spontaneous churning is defined as follows: At each timestep nodes leave the service with a rate linearly increasing with time: *γ* = *μt*/*τ*, reflecting the growing interest in the competing service. We select nodes to leave the service with the probability which was a decreasing function of the node degree (see Supporting Information for details). This was motivated by the fact that more embedded users are more reluctant to leave the site due to exogenous effects.

In summary the model is defined as follows:

1. A network with average degree 〈*k*〉 representing the friendship is created with all users begin member of the OSN.
2. At time *t* users leave with rate *γ* = *μt*/*τ*. (For the reproduction of *k* dependence of *r*_end_ a *k* dependence in node selection may be introduced)
3. Users for which the ratio of the active friends dropped below the threshold *λ* are moved to the *leaving* queue
4. Users in the leaving queue leave definitely with rate *τ*. If this happens check all its friends for threshold

The dynamics defined this way has two timescales: *τ* and 1/*μ*. The latter determines the exogenous timescale and can be fitted to the initial quadratic increase of the number of inactive agents, which relates the model time to real time, therefore it has no influence on the dynamics. The value of the threshold can be adjusted by measuring the peak in the *r*_end_ curves.

We are left with two parameters: *τ* and 〈*k*〉. The timescale *τ* controls the speed of the collapse of the service together with the average degree of the original network. If either *τ* is small (it was zero in the original threshold model^19^) of the network has a high average degree the collapse of the site is almost instantaneous. We found that either large waiting time or low degree is needed to recover the empirical results.

**Figure 7:**
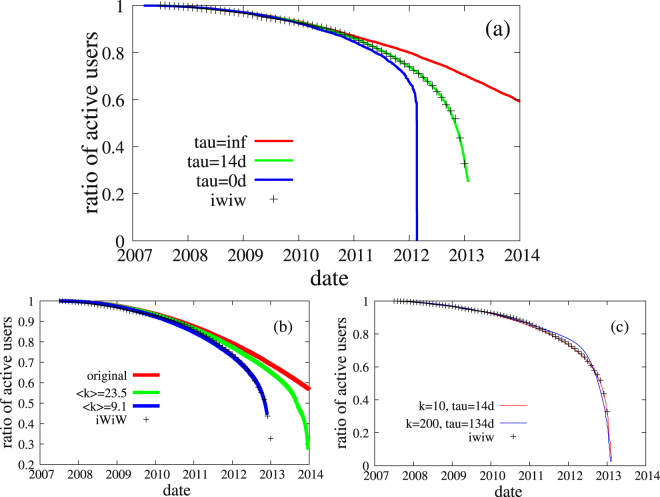
Time evolution of the active users in iWiW. (**a**) The cumulative fraction of active users (all users having a last login date after the indicated time) in iWiW (crosses) and in the cascade model (green). For comparison we show also results with zero characteristic leaving time (blue), and with zero threshold, i.e. no social effect (red). Parameters: *N* = 10000, 〈*k*〉 = 10. (**b**) The cumulative fraction of active users in iWiW and in the model using a county as the underlying network of the model. (**c**) Model fits using random network with average degree 〈*k*〉 = 10, 200.

Fitting our model to the empirical data gives a prefect match as shown in Fig. 7(a) with values of 〈*k*〉 = 10 and *τ* = 14.5 days. Both values are realistic: First, it is natural to assume that people check back for two more weeks after they start getting motivated to leave the service. Second, it was already suggested in^41^ that people have a circle of intimacy of about 12–15 friends and relatives with whom they have regular communication; these are the relationships, which are particularly important for them. In Fig. 7(b) we show the best fit with 〈*k*〉 = 200. It is clear visually that this is a worse fit. For more details see Supporting Information.

Here we used an artificial random network for the model studies. Since the contact network is available from iWiW we may use part of it (the whole being too large for simulation) to study the model. It was shown that political borders inside Hungary are apparent through the contact network^42^ therefore we have decided to use the users in one of the counties to simulate the model.

We have introduced a probability with which the links are kept in order to be able to decrease the degree of the network. After decimating the links we remove nodes with degree 0 and 1. We have studied the evolution of the active users and the results is shown in Fig. 7(c). The best fit was obtained with an average degree 9.1 similar to the model network.

In Fig. 7 we have also shown the time evolution of the ratio of the active users without avalanches and with no waiting time. It can be seen that the time evolution of the three curves depart at middle 2010 where the concurrent service becomes popular and collective effects become important. The model generates cascades similar to those observed in the empirical data, see Fig. 4(b).

A further verification of the model besides Fig. 4 can be obtained by calculating the degree dependence of the number of churned neighbours at leaving the network Fig. 3(a). In order to compare the results of the model with the empirical ones we scaled up the model in the following way: We introduced further (socially less important) links such that the average degree became the same as in the OSN (220). The links were attached to the nodes with a probability proportional to the degree in the small network and the triadic closures were also applied with the same density. The similarity between Fig. 3(a) and (b) are rather convincing.

We have also calculated the average number of churning users as function of the elapsed time. The results match well with the empirical data as shown in Fig. 5.

## Summary

We studied the collapse of an online social network both empirically and by modelling. The empirical results suggest that in the early stage users leave the OSN mostly because they are not anchored to it by a large number of friends, that means, they are not interested enough in online networking or they churn to a competing service. As time goes on, the latter effect seems to dominate resulting in a linearly increasing churning rate following the increasing popularity of the competitor. This erosion of the network due to exogenous influence has the effect that the social pressure becomes critical for an increasing number of active users, who then decide to leave, which leads to a cascading effect and finally, to the collapse of the OSN.

The endogenous and exogenous effects were incorporated in a threshold model, which could reasonably well reproduce the measured quantities of the empirical system. Starting from a recent generalisation^26,32^ of the Watts threshold model^19^ the most important new elements of our model are as follows: First, we have taken into account the endogenous influence by introducing spontaneous churning with a time dependent rate, reflecting the increasing interest in the competitor OSN. Second, we introduced a delay time *τ* between the fulfilment of the threshold criterion and the action under the social pressure. This realistic element was used as a fitting parameter in the model calculations.

The spontaneous churning due to endogenous influence dominates the early phase of the OSN, however, gradually cascades occur and finally they become dominant. From that time any effort to damp spontaneous churning (e.g., by advertisement) is in vain. The apparent stability of the OSN was only due to the relatively large characteristic time for cascading users.

We have found empirically and reproduced by the model that the number of churning neighbours of a churning user within a given time before the event behaves as a power law with a critical time (February 2013, in our case), which can be considered as the collapse time of the service. Similar observations in other systems may be used to predict the date of collapse due to social pressure.

## Methods

### Data

The social network iWiW (4.3 million registered users) was started in 2002 and closed down June 2014. The data used for this study for the period of April 4, 2002–January 23, 2013 includes: date of registration, last login of each user and time stamped link creation information. Using this data we can reconstruct the whole life cycle of iWiW (see Fig. 1). It was at its peak the second most popular site in Hungary^36^ it had 3.5 million active users in 2010, in a country with a population of 10 million (worldwide 13 million native speakers) and at that time about 60% Internet penetration^38^. The site started as a non-profit project in April 2002, however, it was purchased by a giant telecom company in 2006 and remained the leading social network site of Hungary for years; in fact, it was considered as a main driving force behind the speed up of Internet penetration in Hungary.

Till middle 2011 iWiW was invitation based. Every user after 30–50 days of waiting time has got one voucher and new users could register only if they received a voucher from an already existing member. Later vouchers were redistributed irregularly until, in the last period after 2012, the registration became unconditional.

The site has remained widely used even after Facebook became popular worldwide^42^, (see Fig. 2). The story of iWiW came to a sudden end due to various reasons. (i) the introduction of games and application in Facebook made it more attractive especially to young people, (ii) the lack of usable message filtering system made iWiW a prime target of spammers using mainly compromised accounts, (iii) a sizeable (~100 thousand) Hungarians living abroad also gave a strong push to convert friends to Facebook, which rapidly became the prime Hungarian social network site. This resulted in a rapid increase in the number of churning users in 2011 and lead finally to a collapse in 2012. The site was closed down in June 2014 after passing “time capsules” with their network data to the registered users.

The data we used contains anonymised users with registration and last login date, and a time stamped connection list. We used the following data items: timestamp of the last login of a user. The list of friends for each user from the connections which were mutually acknowledged.

In addition to iWiW, our main focus here, we have also studied a much smaller network, available from the Large Stanford Dataset Collection^35^ and which is location based social network site. Gowalla, established in 2007 had ~600.000 users in 2010; it was acquired by Facebook in 2011 and closed down March 2012. The necessary dynamic information to reconstruct the life cycle of the network was available. The results of this network is presented in the Supporting Information.

### Data evaluation

Using the last login timestamp and friendship network Figs 1 and 2(b) were trivially obtained. When counting the number of active friends of a user at the time of its last login we did not consider ordering dates less than 15 days apart. The reason behind this decision was the weekly usage of the site and the similar characteristic time of the churning. In the evaluation of *r*_end_ of the model results we used the same code as for the iWiW data.

Cascade users were defined as number of acquaintances having a last login less than one (two, or four) week(s) before the last login date of the user.

Supplementary movie was made as follows: Movie time goes proportional to real time, Yellow circles indicate the last login of a given user. An arrow is drawn if the head user leaves no later than a week after the last login of the user at the tail. Colour goes from red to light blur with elapsed time.

## Electronic supplementary material

Supplementary Information

Sample cascade
