# Supplementary material for: Cascading collapse of online social networks
Source: Sci Rep. 2017 Dec 1;7:16743. doi: 10.1038/s41598-017-17135-1 (PMC5711899; doi:10.1038/s41598-017-17135-1)
Supplement: Supplementary file 1 — Supplementary Information [file 41598_2017_17135_MOESM1_ESM.pdf]

Supplementary Information to: Cascading collapse of online social networks

János Török[1,2,*] **and János Kertész**[1,2]

[1]Center for Network Science, Central European University, Nádor u. 9, H-1051 Budapest, Hungary
[2]Department of Theoretical Physics, Budapest University of Technology and Economics, H-1111 Budapest, Hungary
[*]torok@phy.bme.hu

ABSTRACT

Other measures on iWiW.

In Fig. 3 (a) of the paper we show the empirical results for the distribution of the fraction of active acquaintances at the time of the last login of users for four different user degrees in iWiW. We measured the position of the maximum for all degrees between 20 and 500. The result is shown in Fig. 1 (a). It can be observed that indeed there is a sharp transition between maximum at $r_{\text{end}} = 0.95$ or around $r_{\text{end}} = 0.45$. The transition point was found to be at around $k \simeq 130$. Thus users having more than 130 acquaintances leave mainly due to social pressure while other with less than 130 friends mainly due to exogenous effects.

We measured how long it takes for the last 5% of one's friends to abandon the site before the user itself also leaves. This 5% was chosen to take in average one intimate friend into account. The resulting average waiting time is plotted in Fig. 1 (b) against the degree of the user. The resulting curve is constant for most of the important degree range of $50<k<1000$ which can be considered realistic. It increases for both low and very large degree users indicating that these users mainly do not leave due to collective effect as their acquaintances on the service do not represent their real social network. The resulting 12 days waiting time is close to the model's fitted waiting time of 14.5 days.

Further details of the model.

Even though the network of the OSN iWiW had an average degree of $\langle k \rangle \simeq 220$ it is obvious that users do not perceive all their acquaintances for continuing to use or abandoning a given service. They are generally interested in their intimate friends with whom they regularly communicate. Thus we have checked which is the average degree for which our model fits best the empirical data. After fitting the exogenous timescale μ for the early (2007−2009) years the waiting time is fitted for the ratio of the active users. Table 1 summarises the results while Fig. 2 (b) shows the best fit with $\langle k \rangle = 10$ and $\langle k \rangle = 200$. It is remarkable that the best fit was found for $\langle k \rangle = 10$. We note that the waiting time which amounts to slightly more than two weeks is also realistic in this case.

We can thus assume that when considering abandoning an OSN users do not care about all their acquaintances but only about their intimate relationships, which amounts to about 10 persons. Considering that on the average 1/3 of the population

$\langle k \rangle$	τ [days]	S
8	9.1	0.003
10	14.5	0.002
14	53.6	0.006
20	67.0	0.011
100	89.4	0.048
200	134.0	0.069

Table 1. Model fitting results: The parameter of the best fit of the model to the iWiW data for different network degrees. μ was fitted to the early parabolic stage so here only a one parameter fit was made for τ. S stands for the least square difference.

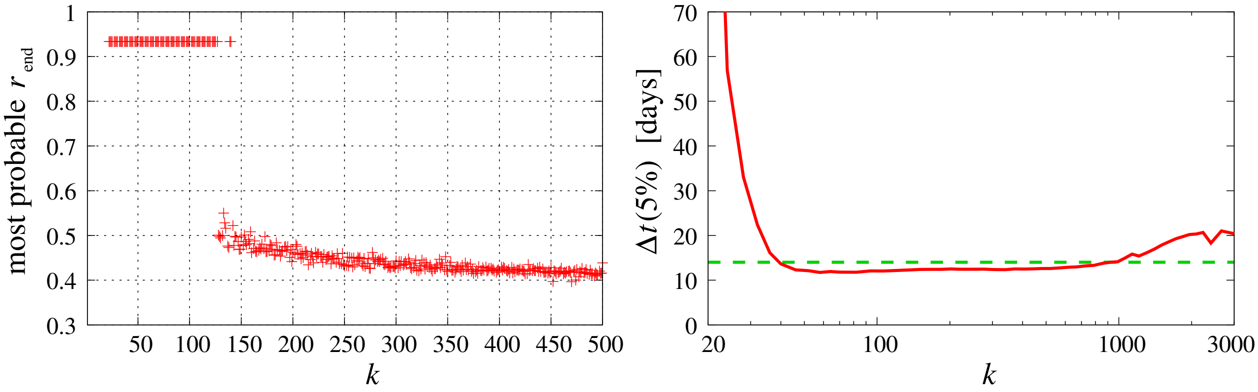

Figure 1. Status of friends just before user is leaving the site. (a) The position of the maximum in the distribution of the fraction of friends that are active at the last login of the user, for different numbers of acquaintances. (b) The average time the last 5% of a user's friend take to abandon the site before the user itself also leaves. The dashed line is the 14.5 days waiting time found in the fit for the inactive users.

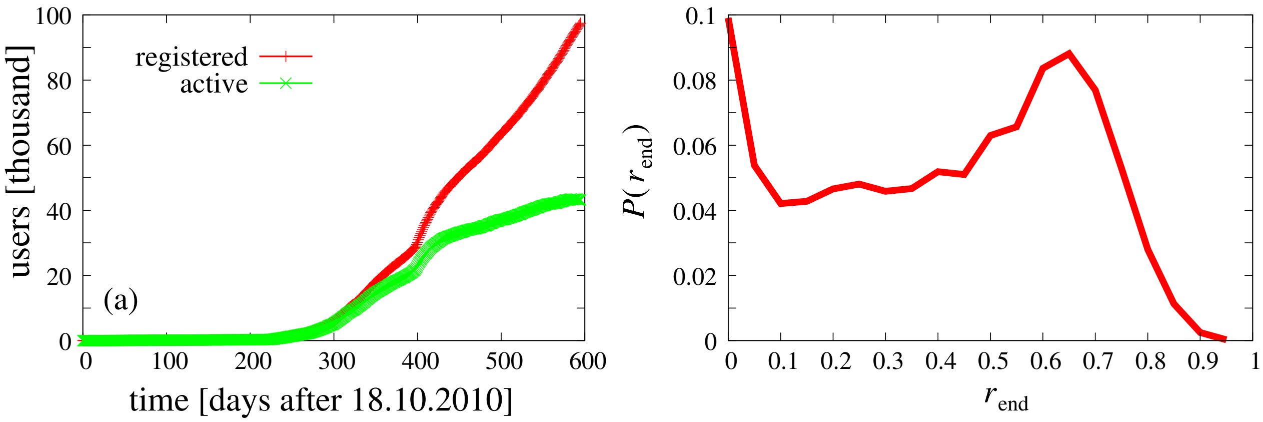

Figure 2. Average user properties in Gowalla.(a) Time evolution of total and active users in (b) The distribution of ratio of active friends at the time of last login for Gowalla.

was not on iWiW, this indicates that people are attached to $12-15$ friends. This number is similar to the size of Dunbar's circle of intimate relationships[5].

We have chosen that around 50% of the links are part of a triangle. This was motivated by the high clustering of the original iWiW data. The model naturally gives similar results without triangle in the network with similar result, but with average degree of 8 and average waiting time of 12 days.

In order to be able to compare our model to the empirical results we extended the sparse network with $\langle k \rangle = 10$ to have average degree of 220 as the original iWiW network. This was done in a probabilistic proportional way (assuming that those who have more intimate friends also have more acquaintances). New edges were added to the network by connecting nodes with probability proportional to their original degree while keeping the ratio of triangles the same as for the sparse network. The extended network was only considered to calculate r_{end} and user selection for removal by exogenous effect it played no role in the cascade process.

For the exogenous effect we defined a selection weight for nodes with different degree: $f(k) \sim k^{-2}$. This favours the selection of the users with low degree for the spontaneous churning as motivated by the observation that low degree nodes leave first. Further motivation for $f(k)$ was taken from the average last login time as function of the node degree. We have tested the sensitivity of the model to the actual form of $f(k)$ but found all results qualitatively similar.

Further empirical network.

In this Section we study another empirical system, the Gowalla, for which the collapse can be analysed. The dataset was obtained from Large Stanford Dataset Collection[6] and is a location based online social network service. Figure 3 (a) shows the

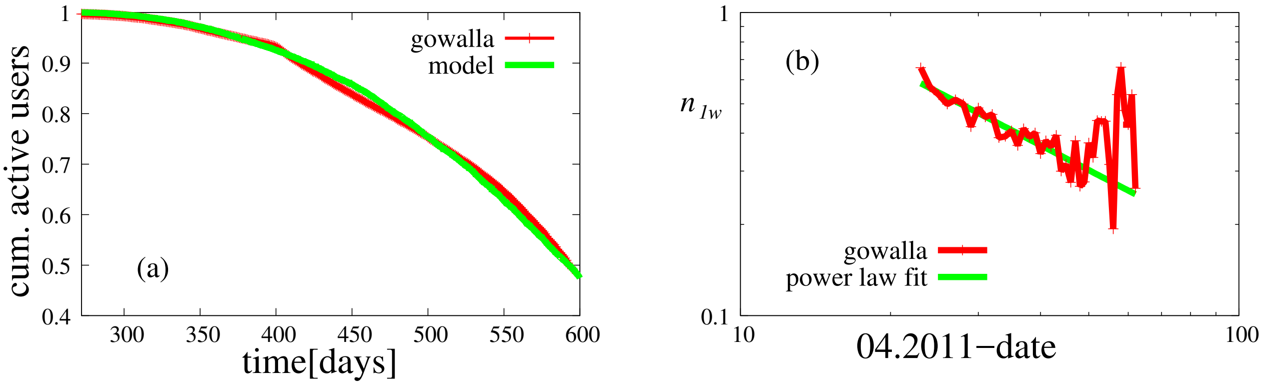

Figure 3. Fit of out model to Gowalla. (a) The cumulative active users for Gowalla and model the relaxation time was more than a year. (b) The number of cascade users as function of the numbur of months prior to 04.2011.

number of registered and active users as function of the time measured in number of days after the foundation. The number of users in Gowalla increased almost linearly, while the number users who left the service began to increase rapidly at around $t = 400$ days, though the new users were still arriving at the same pace. Since friendship network data was available we could calculate the distribution of active friends at the last login of a user. The result is plotted in Fig. 3 (b). For users with intermediate degrees $8-150$ a peak can be seen at around 0.65 which indicates that in case of Gowalla social pressure was important.

The Gowalla social service differs from iWiW as posting locations can be done alone, while activity in an OSN, where the main aim is the interaction with acquaintances, requires more participation of the friends. Consequently for the ratio of active friends at the time of last login we see the other maximum at $r_{\mathrm{end}} = 0$ for Gowalla, indicating that many users who had high affinity in sharing their geolocations keep this habit even if friends stop doing the same.

We have tried to fit the cumulative active users of Gowalla with our model and achieved a reasonable result as shown in Fig. 4 (a), using a similar $\langle k \rangle = 14$ degree network with wider threshold distribution $\lambda = 0.65 \pm 0.25$ and with $\tau = 100$ days waiting time. Let us note that in this case among all users leaving the service only 14% left due to social pressure. This small number of cascade users makes the estimation for the peak of the cascade is rather uncertain. We found 04.2011$\pm$6 months which coincides well with the history of Gowalla, especially in the countries with highest usage (Sweden and Saudi Arabia).

References

1. Suvakov, M., Mitrovic, M., Gligorijevic, V. & Tadic, B. How the online social networks are used: Dialogs-based structure of myspace. *arXiv preprint arXiv:1206.6588* (2012).
2. Tadić, B., Gligorijević, V., Mitrović, M. & Šuvakov, M. Co-evolutionary mechanisms of emotional bursts in online social dynamics and networks. *Entropy* **15**, 5084–5120 (2013).
3. The World Bank. Internet users (1990-2014). URL https://data.worldbank.org/indicator/IT.NET.USER.ZS?locations=HU&page=1.
4. Lengyel, B., Varga, A., Ságvári, B., Jakobi, Á. & Kertész, J. Geographies of an online social network: weak distance decay effect and strong spatial modularity. *PLOS ONE* (2014).
5. Dunbar, R. I., Arnaboldi, V., Conti, M. & Passarella, A. The structure of online social networks mirrors those in the offline world. *Soc. Networks* **43**, 39–47 (2015).
6. Leskovec, J. & Krevl, A. SNAP Datasets: Stanford large network dataset collection. http://snap.stanford.edu/data (2014).
